# Structural Studies and Structure Activity Relationships for Novel Computationally Designed Non-nucleoside Inhibitors and Their Interactions With HIV-1 Reverse Transcriptase

**DOI:** 10.3389/fmolb.2022.805187

**Published:** 2022-02-14

**Authors:** Kathleen M. Frey, Nicole Bertoletti, Albert H. Chan, Joseph A. Ippolito, Mariela Bollini, Krasimir A. Spasov, William L. Jorgensen, Karen S. Anderson

**Affiliations:** ^1^ Department of Pharmacology, Yale University School of Medicine, New Haven, CT, United States; ^2^ Department of Chemistry, Yale University, New Haven, CT, United States; ^3^ Department of Molecular Biophysics and Biochemistry, Yale University School of Medicine, New Haven, CT, United States

**Keywords:** non-nucleoside reverse transcriptase inhibitors, HIV RT, structural studies, computational chemistry, drug design

## Abstract

Reverse transcriptase (RT) from the human immunodeficiency virus continues to be an attractive drug target for antiretroviral therapy. June 2022 will commemorate the 30th anniversary of the first Human Immunodeficiency Virus (HIV) RT crystal structure complex that was solved with non-nucleoside reverse transcriptase inhibitor nevirapine. The release of this structure opened opportunities for designing many families of non-nucleoside reverse transcriptase inhibitors (NNRTIs). In paying tribute to the first RT-nevirapine structure, we have developed several compound classes targeting the non-nucleoside inhibitor binding pocket of HIV RT. Extensive analysis of crystal structures of RT in complex with the compounds informed iterations of structure-based drug design. Structures of seven additional complexes were determined and analyzed to summarize key interactions with residues in the non-nucleoside inhibitor binding pocket (NNIBP) of RT. Additional insights comparing structures with antiviral data and results from molecular dynamics simulations elucidate key interactions and dynamics between the nucleotide and non-nucleoside binding sites.

## Introduction

The HIV-1 (Human Immunodeficiency Virus) is a member of the retroviral family which contains a single-stranded RNA genome and is the major etiological agent involved in the development of acquired immunodeficiency syndrome or AIDS. The World Health Organization now estimates that in 2019 over 40 million people worldwide were infected. The most recent CDC report estimates that in the US over 1.2 million people are infected including about 13% who are unaware of their infections. Over the past decade, the number of people living with HIV has increased, while the annual number of new infections has remained relatively stable. Still, the pace of new infections, at 50,000 per year, continues at far too high a level, particularly among certain socio-economic groups. With the development of antiretroviral therapy (ART), there has been much needed progress over the past decade. Individuals diagnosed with AIDS no longer face a death sentence, but there continues to be a significant need for new therapeutic strategies, new drugs, and new drug combinations to combat this disease. It is imperative to continue to develop new, effective, and safe antiviral compounds in order to stay in front of the HIV virus with its propensity for rapid mutation and resistance. There are several potential targets in the life cycle of the HIV virus including HIV reverse transcriptase (HIV-RT or RT), HIV protease, and more recently viral entry, attachment, and integration (Richman et al., 2009). Drugs targeting RT remain a cornerstone of AIDS therapy as approximately 90% of conventional therapeutic regimens. The drugs that target HIV-1 RT are divided into two classes: nucleoside reverse transcriptase inhibitors (NRTIs) and non-nucleoside reverse transcriptase inhibitors (NNRTIs). The rapid development of drug resistance by the error prone RT, off-target side effects, and issues of viral versus host polymerase selectivity continue to necessitate the discovery of more effective NRTIs and NNRTIs with improved safety, pharmacological, and drug resistance profiles.

The first three-dimensional (3D)-structure of RT was solved by the Steitz lab in 1992. This structure was solved in the presence of the NNRTI, nevirapine (Kohlstaedt et al., 1992). The 3-D structure revealed that nevirapine was bound in an allosteric binding pocket 10-Å away from the RT polymerase active site, which is absent in the apo form of RT (Clark et al., 1995; Rodgers et al., 1995). NNRTIs significantly inhibit the catalytic function of RT by binding in the non-nucleoside inhibitor binding pocket (NNIBP). Previous kinetic studies have shown that NNRTIs act as non-competitive inhibitors by slowing the polymerization reaction (Hopkins et al., 1989; Spence et al., 1995b; Rittinger et al., 1995; Nissley et al., 2007).

A major clinical concern in the use of these drugs is the development of resistant strains of HIV harboring mutations in RT (de Bethune, 2010; Iyidogan and Anderson, 2014). For NNRTIs, among the most common resistance mutations are Lys103Asn (K103N) and Tyr181Cys (Y181C), which are observed in 57 and 25% of patients failing treatment (Iyidogan and Anderson, 2014). In particular, Y181C has been a major hurdle in development of NNRTIs (Bollini et al., 2011; Jorgensen et al., 2011; Gubernick et al., 2016); it renders many NNRTIs including nevirapine ineffective. Moreover, nevirapine remains on the World Health Organization (WHO’s) Model List of Essential Medicines which is used as a single agent to prevent mother-to-child viral transmission. The double variant Y181C/K103N abrogates efficacy for all NNRTIs except those most recently introduced etravirine, rilpivirine, and doravirine (Janssen et al., 2005; Iyidogan and Anderson, 2014; Martin et al., 2020). More recently, mutations at K101 including K101P have been reported to be problematic with these newer generation NNRTIs including rilpivirine (Balamane et al., 2012). In addition, rilpivirine has very low aqueous solubility and potential cardiotoxicity liabilities due to inhibition of the HERG ion channel that limit dosing regimens (Kudalkar et al., 2018).

Over the past several years, our research has focused on developing improved NNRTIs relative to current FDA approved NNRTIs such as rilpivirine. This includes better pharmacological properties including enhanced aqueous solubility, lack of inhibition on HERG ion channel as well as efficacy on drug resistant HIV variants including Y181C, Y181C/K103N, and K101P. Our strategy combines state-of-the-art technology for *in silico* virtual screening/structure-based drug design, synthetic organic chemistry, mechanistic enzymology and protein crystallography, and pharmacological assays (Kudalkar et al., 2017; Kudalkar et al., 2018). In the current study, we describe the structure activity relationship and structural analysis of some of these NNRTIs and their interactions with HIV-1 RT. Analysis of the crystal structures, paired with antiviral data and molecular dynamics (MD) simulations, elucidate key interactions between the NNRTI and residues in the NNIBP.

## Materials and Methods

### Protein Expression and Purification

RT52A construct expression for RT (WT), RT (Y181C) and RT (K103N/Y181C) were expressed recombinantly in *E. coli* strain BL21(DE3) using methods described previously (Frey et al., 2015). Recombinant RT was purified using cobalt IMAC followed by removal of the N-terminal 6x-histidine tag *via* overnight cleavage with HRV protease. The collected sample was then further purified using ion exchange chromatography. Purified sample was flash frozen with liquid nitrogen until needed for crystallization.

### Protein Crystallization, Data Collection, and Refinement

Chemical synthesis for all compounds crystallized with RT enzymes except compound **1** (vide infra) have been reported previously (Frey et al., 2012; Frey et al., 2015). Crystals of RT were prepared using co-crystallization and/or soaking methods described previously. The final optimized condition for crystal growth consisted of 18% (w/v) PEG 8000, 100 mM ammonium sulfate, 15 mM magnesium sulfate, 5 mM spermine, and 50 mM citric acid, pH 7.5. Crystals were transferred to a cryo-solution containing 27% (v/v) ethylene glycol and flash-cooled with liquid nitrogen.

Diffraction datasets for all crystals were collected at Brookhaven NSLS on beamline X29A and APS on beam line 24-ID-E through NE-CAT. Diffraction data was scaled and merged in space group C2 using HKL3000 (Minor et al., 2006). Difference Fourier methods or molecular replacement was used to determined phases using the program Phaser (McCoy et al., 2007). Models for all RT complexes were built into the electron density using program Coot (Emsley et al., 2010) followed by refinement using Phenix Refine (Adams et al., 2010). Several iterations of refinement continued until acceptable R-values and geometric parameters were achieved. All structures were validated using Molprobity and Ramachandran plots (Chen et al., 2010). Structures were analyzed using molecular viewer Pymol (Schrödinger, 2021b). Iterative build omit *σ*_A_-weighted 2*mF*_0_—*F*_*c*_ electron density maps were generated using Phenix Autobuild (Terwilliger et al., 2008).

### Molecular Dynamics Simulation

Crystal structures for compound **1**, compound **2**, RT:dsDNA:dCTP (PDB code: 6P1I), and RT:dsDNA:(-)3TC-TP (PDB code: 6OUN) complexes were used for molecular dynamics (MD) simulations and analysis. All structures were prepared by adding hydrogens, assigning charges, capping the termini, and deleting non-interacting water molecules using the Protein Preparation tool in Maestro (Schrödinger, 2021a). The Protein Preparation tool is a collection of programs that prepare biomolecular models for modeling calculations. Prepared models were used to build the system for MD. The models were energy minimized prior to simulation.

Using the System Builder in Desmond (Schrödinger, 2021a), we predefined the solvent model to be TIP4P, calculated the box volume, and then minimized the structure. The system was neutralized by the addition of Mg2^+^ and Cl^−^ ions, simulating a concentration of 0.15 M. The force field applied to each system was OPLS within the System Builder in Desmond. Water molecules and ions around the enzyme were built into the system within the calculated orthorhombic box volume for soluble proteins. The average calculated box volume = 1,337,030 Å^3^. MD using Desmond (Schrödinger, 2021a) was performed for each model system. The models were relaxed before the simulation. The canonical ensemble or NVT was applied using the Berendsen thermostat (temperature = 300°K) and Berendsen barostat (pressure = 1.01325 bar). Simulation time was set to 24 nanoseconds (ns) to allow for system equilibration. A total of 1000 snapshots (24 picoseconds/interval) were generated for each RT:NNRTI complex. Trajectories from each simulation were analyzed using Simulation Interaction Analysis and Simulation Event Analysis programs in Desmond (Schrödinger, 2021a). Simulations were analyzed for equilibration and convergence by examining root mean square deviation (RMSD) versus simulation time plots.

### Antiviral Data and Synthesis for Compound 1

The antiviral efficacy of compound **1** was evaluated in MT-2 cells infected with HIV-1 as previously described (Lee et al., 2014; Lee et al., 2015; Kudalkar et al., 2017). Briefly, the antiviral activities against the IIIB strain of HIV-1 were measured using human lymphoid MT-2 T-cells; The antiviral efficacy (EC_50_) values are obtained as the dose required to achieve 50% protection of the infected cells by the MTT colorimetric method (Pannecouque et al., 2008). Simultaneously, the cytotoxicity (CC_50_) values for inhibition of growth of MT-2 cells in the absence of virus are determined. The analyses used triplicate samples at each concentration and typically at least two biological replicates. Representative data for compound **1** is shown in (Supplementary Figure S11).

The synthesis and characterization data for compound **1** is described in Supplementary Material. The synthesis and antiviral activities of compounds **2**-**4** have previously been reported (Lee et al., 2014; Lee et al., 2015). A summary of the antiviral activities and aqueous solubilities is reported in Table 1.

**TABLE 1:** Antiviral data and solubility measurements for Compounds 1-4.

Cmpd	EC_50_ WT (μM)	CC_50_ WT (μM)	EC_50_ Y181C (μM)	CC_50_ Y181C (μM)	EC_50_ K103N/Y181C (μM)	CC_50_ K103N/Y181C (μM)	Solubility (µg/ml)
(**1**)	1.1	>100	N/A	>100	N/A	>100	N/D
(**2**)^a^	0.0012	4.5	<0.1	3.4	<0.1	2.4	14.2
(**3**)^b^	0.0011	>100	0.008	>100	0.006	>100	9.1
(**4**)^a^	0.001	10.1	0.00057	8.1	0.039	8.5	8.16

Compd = compound; N/A = not active; N/D = not determined.

a (Lee et al., 2015).

b (Lee et al., 2014).

## Results

**FIGURE 1:**
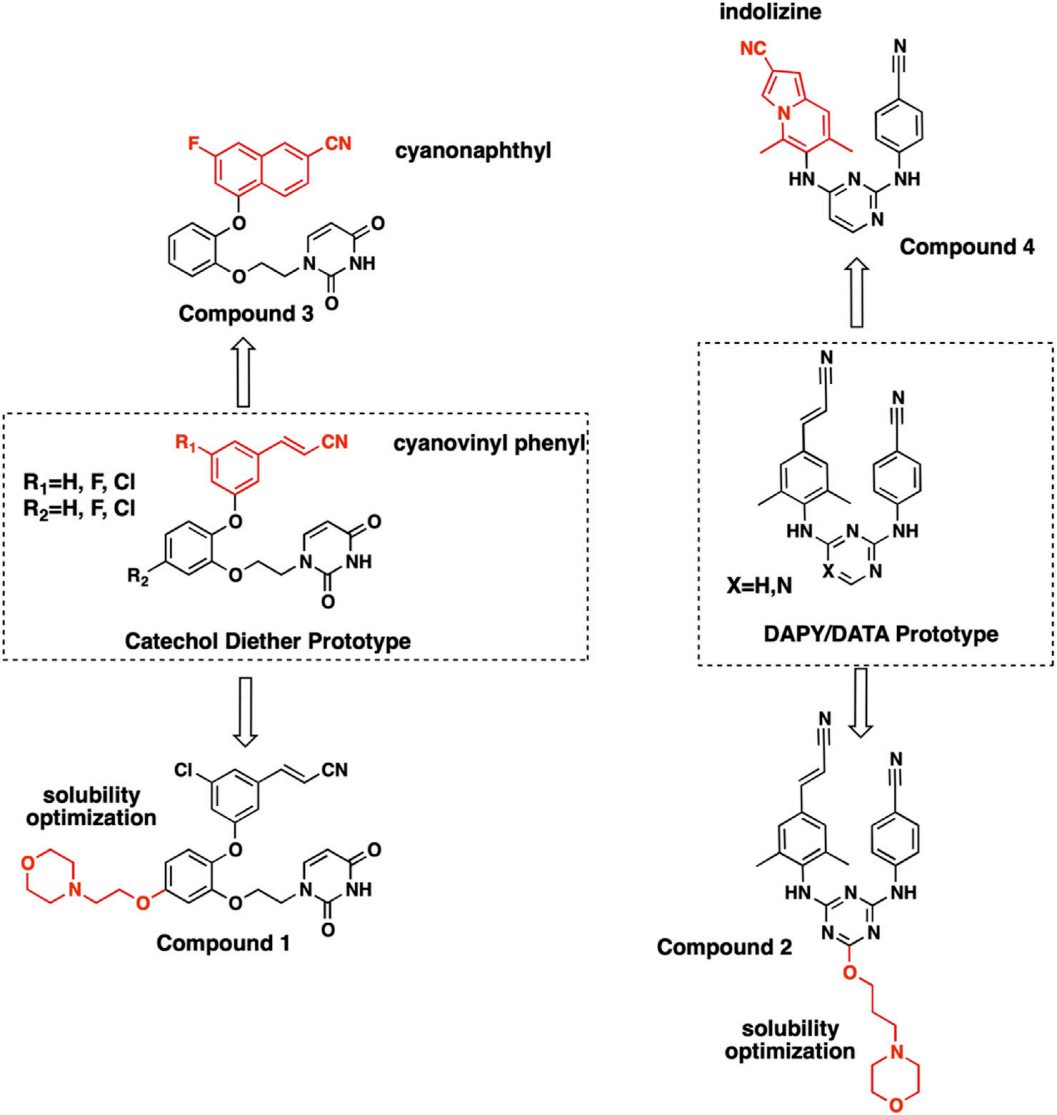
Structures of NNRTI compounds co-crystallized with RT and evaluated for SAR. Synthesis and characterization for compound **1** is provided in Supplementary Material. Synthesis and characterization for compounds 2-4 have been reported previously (Lee et al., 2014; Lee et al., 2015).

For several years, we have been designing NNRTIs targeting variants of RT. Figure 1 depicts the various compounds designed to target RT and variants. New antiviral data for compound **1** along with 7 new crystal structures help elucidate structure activity relationships (SAR) for major compound classes. The following vignettes summarize our design of these various chemical classes of NNRTIs that possess antiviral activity.

### General Structure Details for RT Complexes

**FIGURE 2:**
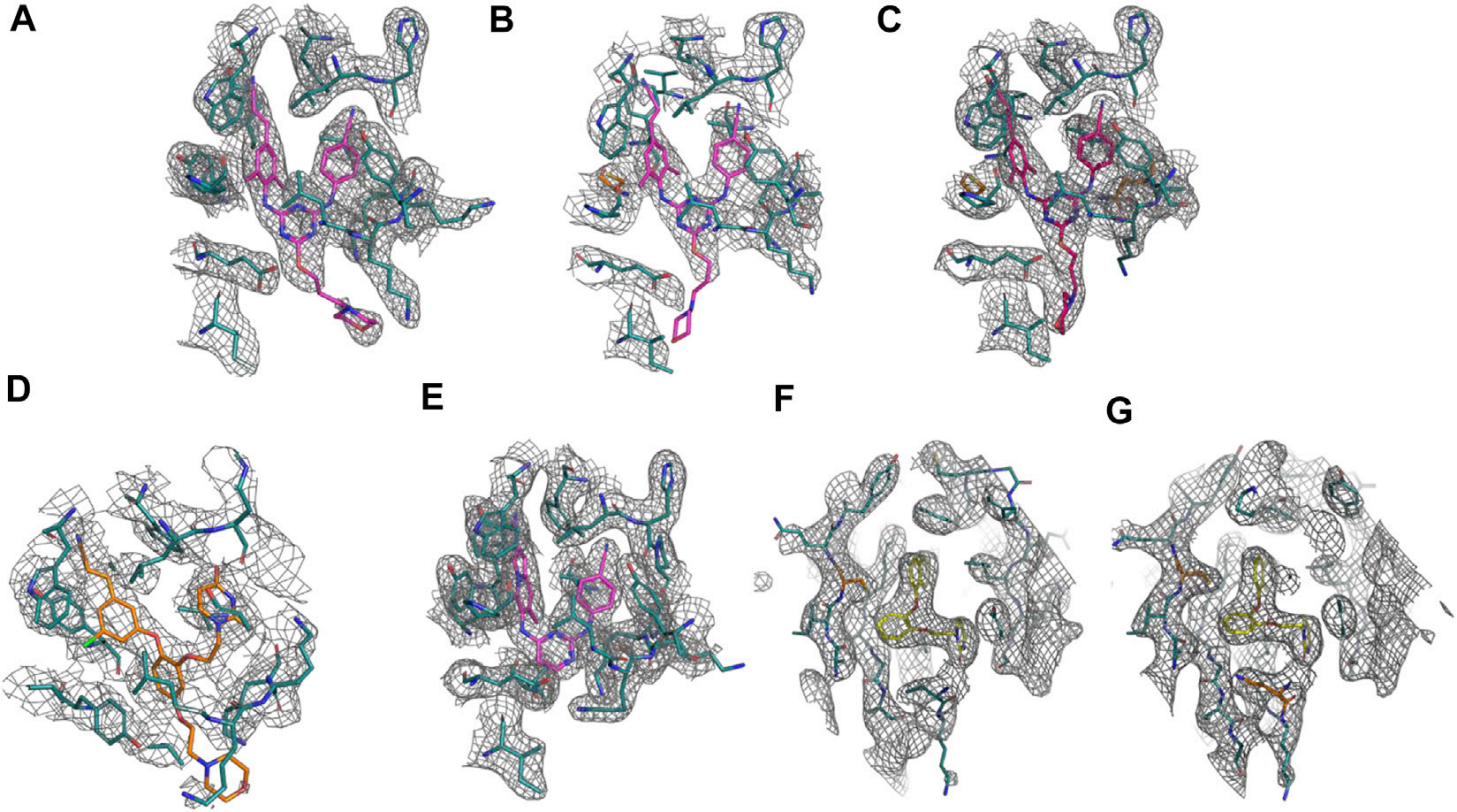
Omit electron density of the non-nucleoside inhibitor binding site for all crystal structure complexes: **(A)** RT (WT):**2**. **(B)** RT (Y181C):**2**. **(C)** RT (K103N/Y181C):**2**. **(D)** RT (WT):**1**. **(E)** RT (WT):**4**. **(F)** RT (Y181C):**3**. **(G)** RT (K103N/Y181C):**3.** Omit electron density maps were generated using simulated annealing omit build in Phenix (Adams et al., 2010).

We determined crystal structure complexes for wildtype (WT) and variants of RT with various NNRTI compounds. Resolution for the collected crystal structures ranged from 2.368–3.089 Å. Data collection and refinement statistics for all RT complexes are listed in Tables 2, 3. The electron density defines the compounds and all interacting residues for all RT complexes Figure 2. The compounds co-crystallized bind the NNIBP causing RT to adopt an “open” conformation commonly observed for RT NNRTI complexes (Das et al., 2008; Lansdon et al., 2010; Frey et al., 2015).

**TABLE 2:** Data collection and refinement statistics for RT crystal structures in complex with compound **2**.

Structure	RT(WT):2	RT(Y181C):2	RT (K103N/Y181C):2
**PDB Code**	**7SO1**	**7SO2**	**7SO3**
**Data Collection statistics**			
Wavelength	1.0	1.0	1.0
Resolution Range	35.58–2.727	42.88–3.089	37.86–2.767
Space Group	C 1 2 1	C 1 2 1	C 1 2 1
Unit Cell: Dimensions, Angles	a = 162.03, b = 73.4, c = 108.525	a = 161.105, b = 73.101, c = 107.82	a = 162.597, b = 73.87, c = 108.435
	α = 90, *β* = 100.375, *γ* = 90	α = 90, *β* = 99.629, *γ* = 90	α = 90, *β* = 101.558, *γ* = 90
Unique Reflections	33,088	22,651	31,488
Multiplicity (Redundancy)	3.7	3.6	3.5
Completeness, %	98.18 (90.69)	98.57 (89.51)	97.08 (91.25)
I/sigma	14.9 (2.8)	26.9 (2.2)	25.5 (1.9)
Wilson B-factor	59.87	97.42	73.68
Highest Shell	0.128 (0.296)	0.093 (0.435)	0.113 (0.483)
**Refinement Statistics**			
R-free	0.2690	0.2879	0.2754
R-work	0.2254	0.2317	0.2291
Average B-factor	70.94	98.19	77.61
RMSD Bonds (Angles)	0.003 (0.70)	0.003 (0.69)	0.004 (0.89)
Ramachandran: Favored, Allowed, Outliers	95.48, 3.79, 0.74	93.97, 5.5, 0.54	97.94, 2.06, 0
Clashscore	4.14	5.24	4.65
Number of Atoms: Protein, Ligands, Solvent	7828, 38, 0	7599, 38, 0	7591, 38, 4

**TABLE 3:** Data collection and refinement statistics for RT (WT) crystal structures in complex with compounds 1 and 4, RT (Y181C) with compound 3, and RT (K103N/Y181C) with compound **3**.

Structure	RT(WT):1	RT(WT):4	RT (Y181C):3	RT (K103N/Y181C):3
**PDB Code**	**7SNP**	**7SNZ**	**7SO4**	**7SO6**
**Data Collection Statistics**				
Wavelength	1.0	1.0	1.0	1.0
Resolution Range (Highest Shell)	35.597–2.89 (2.95–2.89)	42.830–2.368	37.98–2.946 (3.12–2.946)	41.46–2.793 (2.873–2.793)
Space Group	C 1 2 1	C 1 2 1	C 1 2 1	C 1 2 1
Unit Cell: Dimensions, Angles	a = 224.82, b = 69.048, c = 104.337	a = 161.909, b = 75.527, c = 108.95	a = 161.3, b = 73.842, c = 107.063	a = 161.74, b = 74.17, c = 108.6
	*α* = 90, *β* = 106.292, *γ* = 90	*α* = 90, *β* = 100.458, *γ* = 90	*α* = 90, *β* = 100.12, *γ* = 90	*α* = 90, *β* = 99.369, *γ* = 90
Unique Reflections	34,585	50,672	26,126	31,556
Redundancy (Highest Shell)	3.8 (3.7)	3.7 (3.6)	—	—
Completeness, %	99.43 (99.83)	99.68 (97.39)	98.75 (91.93)	99.31 (94.92)
I/sigma	17.40 (1.9)	25.85 (1.81)	17.82 (2.41)	14.51 (2.62)
Wilson B-factor	83.81	52.40	82.38	74.23
R-merge (Highest Shell)	0.100 (0.588)	0.098 (0.488)	0.053 (0.663)	0.073 (0.571)
**Refinement Statistics**				
R-free	0.2769	0.2460	0.2716	0.2749
R-work	0.2302	0.2097	0.2284	0.2383
Average B-factor	61.86	59.81	89.17	88.75
RMSD Bonds (Angles)	0.005 (0.95)	0.005 (0.94)	0.002	0.003
Ramachandran: Favored, Allowed, Outliers	94.45, 5.34, 0.21	98.52, 1.27, 0.21	96.87, 3.13, 0	97.46, 2.54, 0
Clashscore	4.14	5.24	3.51	3.98
Number of Atoms: Protein, Ligands, Solvent	7828, 38, 0	7599, 38, 0	7670, 36, 3	7520, 32, 14

### Structure Activity Relationships for Novel NNRTIs

Extensive SAR has been conducted to design novel NNRTIs with improved resistance profiles and optimal physiochemical properties (Bollini et al., 2011; Jorgensen et al., 2011; Lee et al., 2013; Lee et al., 2014; Lee et al., 2015; Chan et al., 2017; Ippolito et al., 2021). Through each iteration of design, new insights were gained regarding key interactions with RT and the NNIBP. The following specific insights led us to develop some of the most potent NNRTIs with improved drug-like properties to date as compared to the current FDA approved NNRTIs as well as others previously published (De Clercq, 1998; De Clercq, 2007; De Clercq, 2010).

#### DAPY/DATA Versus Catechol Diether

Diarylpyrimidine (DAPY) and diaryltriazine (DATA) SAR led to the development of NNRTIs etravirine and rilpivirine, 2 flexible DAPY NNRTIs that remain potent for RT variants (Das et al., 2008; Lansdon et al., 2010). While the DAPY pharmacophore retained interactions with RT variants, solubility remained a major limitation. The development of the catechol diether system with a terminal uracil resulted in NNRTI compounds with picomolar potency and increased solubility (Bollini et al., 2011). Further optimization focused on improving potency for major RT variants RT (Y181C) and RT (K103N/Y181C) in addition to increasing solubility (Bollini et al., 2011; Bollini et al., 2013a; Bollini et al., 2013b; Frey et al., 2015).

The NNIBP consists of several hydrophobic residues requiring complementary nonpolar NNRTIs. Consequently, solubility for some of the most potent NNRTIs remains a major limitation. In order to improve aqueous solubility, ether-linked morpholine groups were added to DATA and catechol diether systems (Lee et al., 2013). The addition of an ethoxy morpholine to catechol diether compound **1** significantly reduced potency for RT (WT). The attachment of a longer propoxy morpholine to DATA compound **2** retained potency for RT (WT), RT (Y181C), and RT (K103N/Y181C) variants.

**FIGURE 3:**
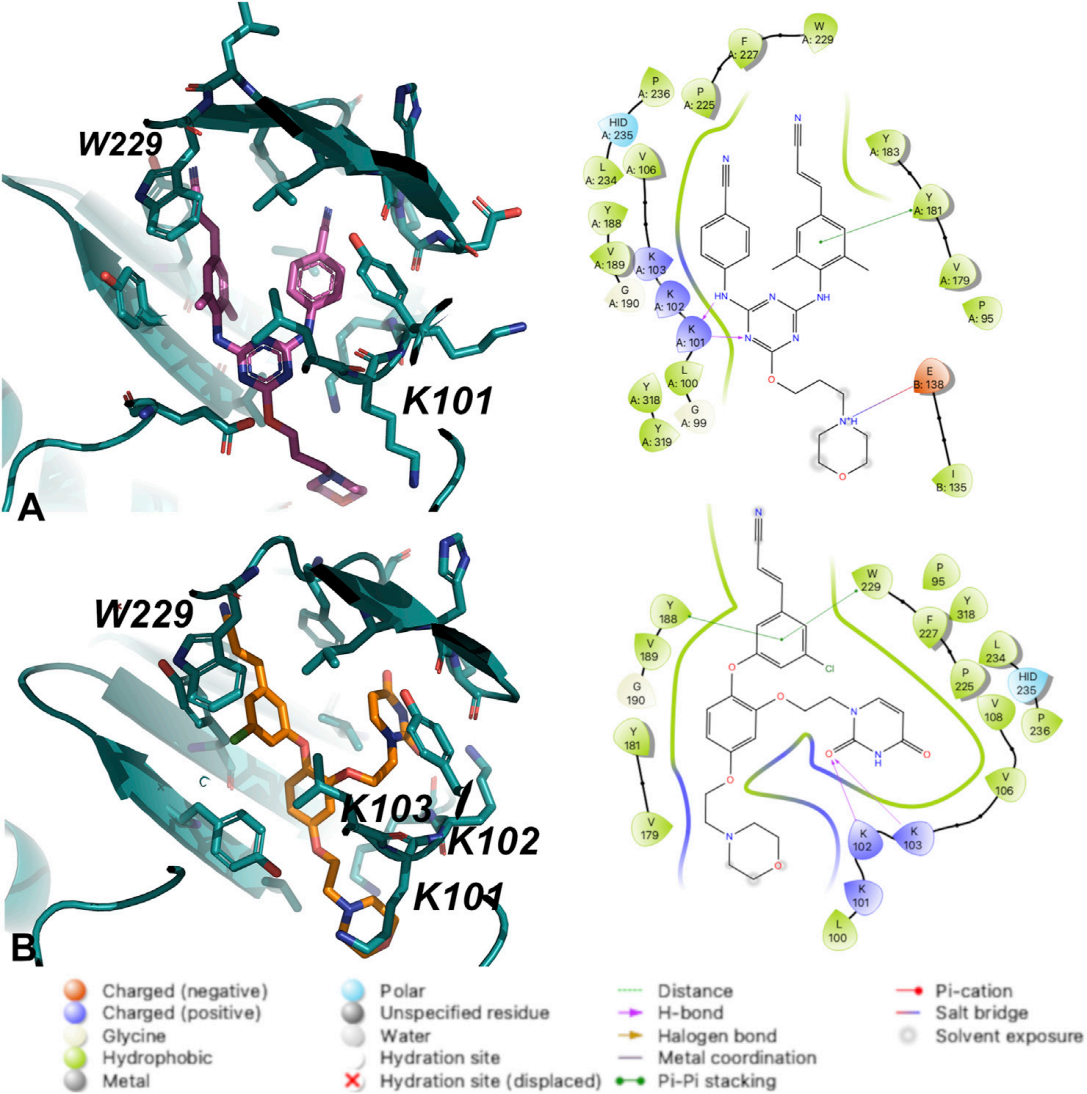
Interactions between **(A)** Compound **2** and **(B)** Compound **1** with residues in the NNIBP of RT (WT). 3D depiction of interactions (left) and 2D schematic diagram of interactions (right). Hydrogen bonds are represented by purple arrows; *π-π* interactions are represented by green lines between aromatic rings and residues; van der Waals contacts are made between all listed residues within a radius of 4 Å. The key provided specifies residue type, water molecules, and interactions.

Crystal structures for RT (WT) in complex with both **1** and **2** and were determined and analyzed to explain the difference in potency. Compound **2** appears to make several van der Waals contacts with residues in the NNIBP. Compound **2** also hydrogen bonds with K101 *via* the 1) backbone CO and aniline linker; and 2) the backbone NH and the triazine N. Key interactions between RT (WT) and **1** include hydrogen bonds between the uracil carbonyl and 1) the N side chain of K102; and 2) the backbone NH of K103. Additional π-π stacking interactions were observed between Y188 and W229 with compound **1** along with several van der Waals contacts (Figure 3). Based on the analysis from crystal structures, the interactions between compounds **1** and **2** are similar and it remained unclear why **2** retained potency for RT (WT), RT (Y181C) and RT (K103N/Y181C).

**FIGURE 4:**
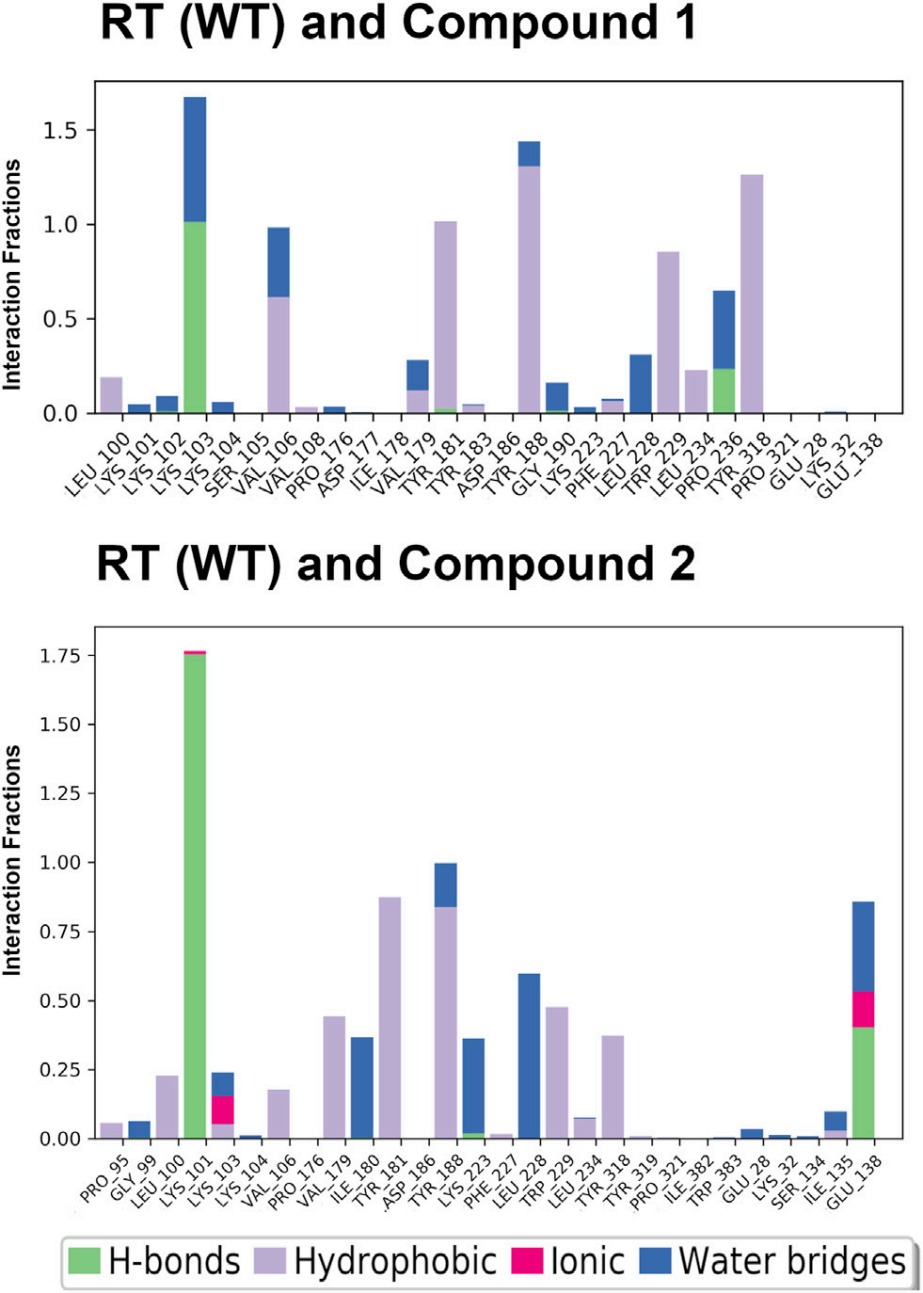
Interaction fractions for RT (WT) and compounds **1** and **2** in the 24 ns simulations. Protein interactions with compounds **1** and **2** were monitored throughout the simulation and are characterized by hydrogen bonds (H-bonds; green); hydrophobic contacts (purple); ionic bonds (pink), and water bridges (blue). Simulation interactions diagrams provided in Supplementary Material (Supplementary Figures S3, S6) show % interactions between specific residues and functional groups of the compounds. The interaction fraction describes how long interactions are maintained over the course of the trajectory snapshots. An interaction fraction of 1.0 suggests that this interaction is maintained in the simulation 100% of the time. Interactions fractions >1.0 represent residues capable of making multiple contacts with the compound.

The crystal structures could not provide an explanation for why compound **1** has reduced affinity for RT (WT). In order to explore this more, we conducted MD for RT (WT) crystal structures in complex with compound **1** and compound **2**. Results from a 24 ns simulation identified key interactions maintained between ether-linked morpholine compounds **1** and **2**. Compound **2** maintains stronger contacts with K101 and E138 (observed in >100% of snapshots due to multiple bonds with K101), whereas compound **1** only maintains one strong hydrogen bond with K103 **(** Figure 4; Supplementary Figures S3, S6 **)**. The propoxy-linked morpholine in compound **2** is longer and makes additional ionic and hydrogen bonds with E138. The ethoxy-linked morpholine in compound **1** does not make any strong contacts with E138 or residues in the binding site. The salt bridge formed between K101 and E138 has been associated with stabilizing NNRTI binding in the NNIBP (Das et al., 2008). In the case of compound **2**, the addition of the propoxy morpholine disrupts the salt bridge interaction but permits an additional contact with E138.

In addition to the stabilizing hydrogen bond, compound **2** maintains more stronger contacts with residues during the simulation **(** Figure 4 **).** Compound **3** makes more van der Waals contacts with hydrophobic residues such as P95, L100, V106, V179, Y181, Y188, W229, L234, Y318, and I135. A π-π interaction between the cyanovinylphenyl of compound 2 and W229 is also apparent.

#### Cyanovinyl Phenyl to Cyanonaphthyl/Cyanoindolizine

**FIGURE 5:**
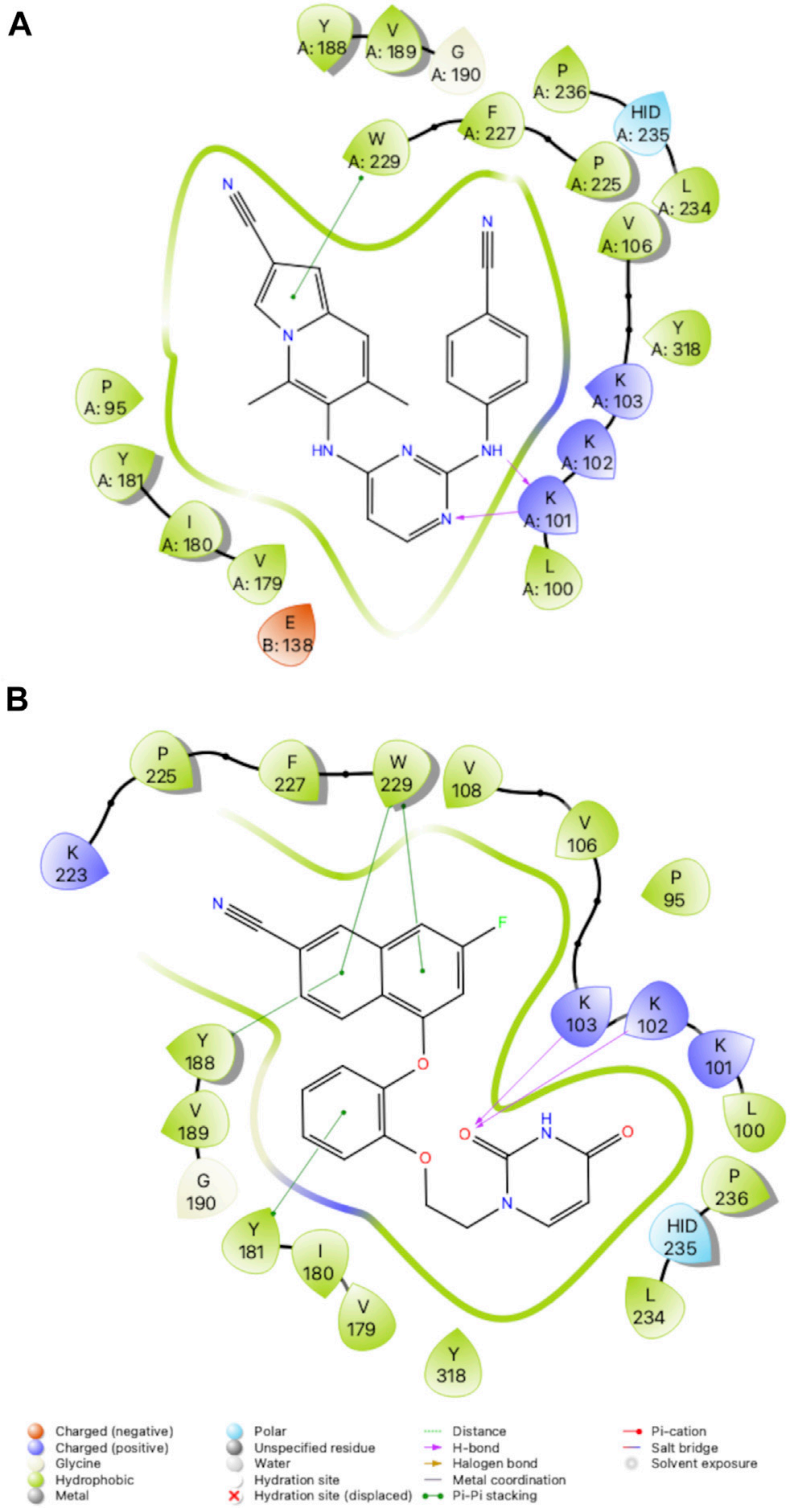
2D Interaction diagrams for RT complexes with compound **4** **(A)** and compound **3** **(B)**. Hydrogen bonds are represented by purple arrows; *π-π* interactions are represented by green lines between aromatic rings and residues; van der Waals contacts are made between all listed residues within a radius of 4 Å. The key provided below specifies residue type, water molecules, and interactions.

Previous work conducting SAR for DAPYs/DATAs and catechol diether compounds concluded that the CN-vinyl phenyl moiety was important for π-π interaction and van der Waals contacts with aromatic residues Y181, Y188, and W229 (Frey et al., 2012; Frey et al., 2015). Concern for unwanted Michael additions to the cyanovinyl group prompted the design of new derivatives that replaced the CN-vinyl with naphthyl and indolizine aromatics. Replacement of the CN-vinyl phenyl with a CN-naphthyl in the catechol diether series resulted in compounds such as compound **3** (Figure 2). The addition of the naphthyl in compound **3** enhances edge-to-face π-π interactions with residues Y188 and W229; each benzene ring in the naphthyl can interact with the residues as opposed to the single phenyl (Figure 5). For the DAPY series, replacing the CN-vinyl phenyl with an CN-indolizine in compound **4** increases edge-to-face π-π interaction with W229 (Figure 5). The pyrimidine maintains hydrogen bonding with Lys101 and several van der Waals contacts.

**FIGURE 6:**
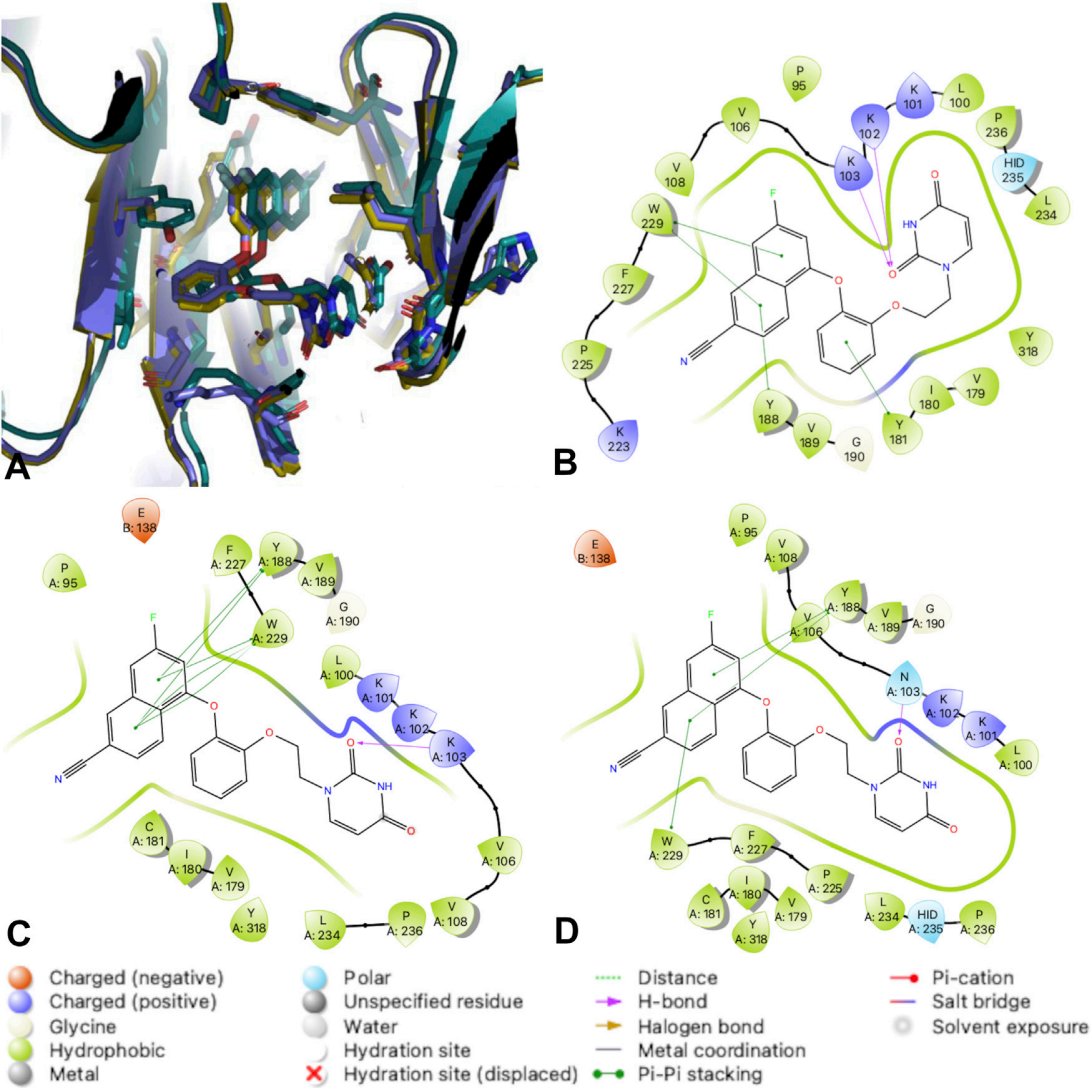
RT complexes with compound 3. **(A)** An overlay of RT (WT) (teal); RT (Y181C) (lavender); and RT (K103N/Y181C) (gold) in complex with compound 3. **(B)** 2D interaction diagram for RT (WT) in complex with compound 3. **(C)** 2D interaction diagram for RT (Y181C) in complex with compound 3. **(D)** 2D interaction diagram for RT (K103N/Y181C) in complex with compound 3. Hydrogen bonds are represented by purple arrows; *π-π* interactions are represented by green lines between aromatic rings and residues; van der Waals contacts are made between all listed residues within a radius of 4 Å. The key provided specifies residue type, water molecules, and interactions.

Compound **3** also retains potency for RT (Y181C) and RT (K103N/Y181C) variants as shown in the antiviral data (Table 1). Analysis of the crystal structures suggest that many key interactions are maintained with residues in the NNIBP (Figure 6). Most notably, π-π interactions with residues Y188 and W229 are retained in crystal structures of compound **3** and RT (Y181C) and RT (K103N/Y181C) variants. The key π-π interaction along with the maintained hydrogen bond with K/N103 results in potent nanomolar activity for both variants.

#### SAR for Variants RT (Y181C) and RT (K103N/Y181C)

**FIGURE 7:**
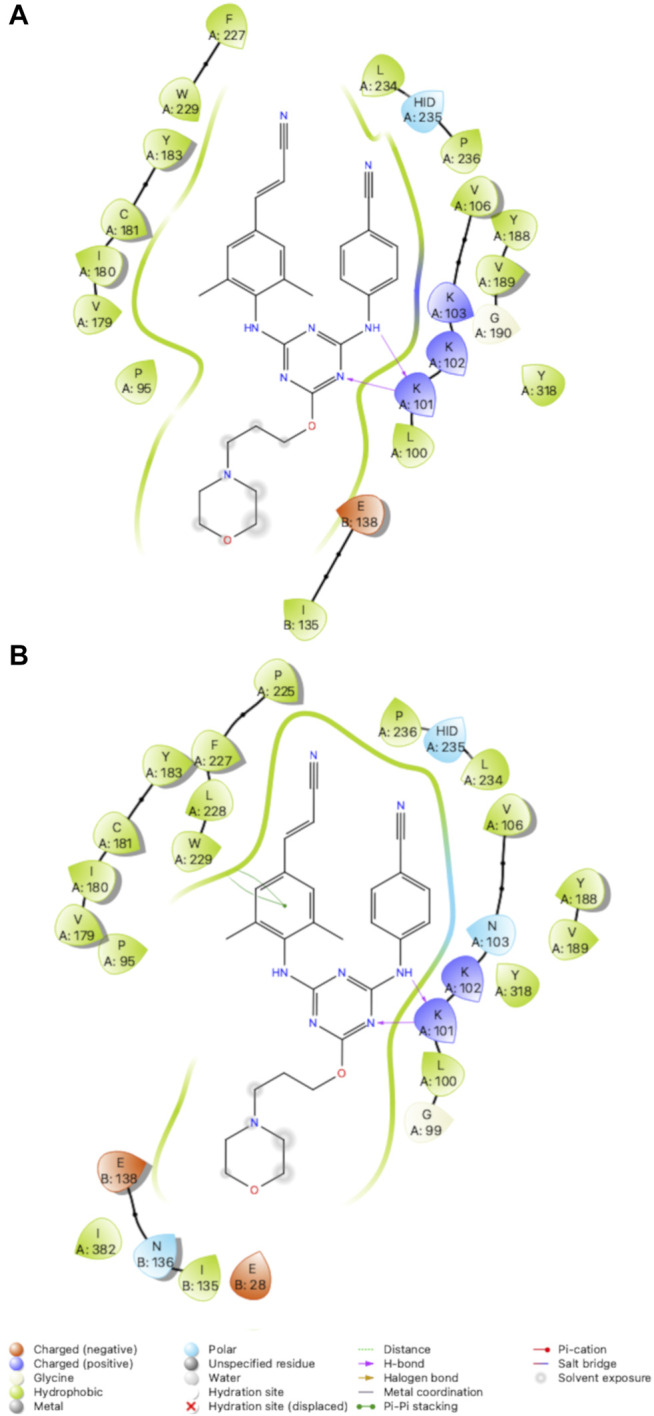
2D Interaction diagrams for RT (Y181C): Compound **2** **(A)** and RT (K103N/Y181C): Compound **2** **(B)**. Compound 2 retains hydrogen bonds with K101 and van der Waals contacts with several residues for both variants. Hydrogen bonds are represented by purple arrows; *π-π* interactions are represented by green lines between aromatic rings and residues; van der Waals contacts are made between all listed residues within a radius of 4 Å. The key provided below specifies residue type, water molecules, and interactions.

Compound **2** has increased solubility compared to other compounds tested (solubility = 14.2 μg/ml and maintains affinity for Y181C and K103N/Y181C variants of RT. Table 1 reports solubility for compounds **2-4** in addition to antiviral data for variants RT (WT), RT (Y181C) and RT (K103N, Y181C. We compared 3 crystal structures of compound **2** in complex with RT (WT), RT (Y181C), and RT (K103N/Y181C) to examine interactions that promote affinity for Y181C and K103N/Y181C variants. Key interactions such as the 2 hydrogen bonds with K101 are maintained in the RT (Y181C) and RT (K103N/Y181C) crystal structures (Figure 7). In both RT (Y181C) and RT (K103N/Y181C) structures, the absence of Y181 causes the DATA scaffold to shift toward aromatic residues Y183, Y188, and W229 to make van der Waals contacts. Antiviral data also complements the analysis from the structures as compound **2** retains nanomolar potency for the virus containing wild-type RT and variants with Y181C and K103N/Y181C mutations (Table 1; WT: 0.0012 µM; Y181C: 0.012 µM Y181C/K103N: 0.0013 µM). (Lee et al., 2014; Lee et al., 2015)

### MD Simulation to Understand NRTI and NNRTI Effects on Polymerase Activity

NNRTIs have a different mechanism of action than NRTIs that compete with nucleotides for the active site and DNA incorporation. Several kinetic and structural studies have shown that NNRTIs slow the rate of polymerization by causing a conformational change in RT upon binding the allosteric NNIBP (Spence et al., 1995b) Kinetic studies have also shown coordination between the polymerase active site and NNIBP, where NNRTIs enhance the binding of NRTIs (Spence et al., 1995a; Rittinger et al., 1995).

In order to compare the global movement within NRTI/DNA complexes, we conducted additional MD simulations for a catalytic complex of RT bound to a double-stranded DNA primer-template and 1) dCTP (PDB code: 6P1I) and NRTI (-)-3TC (PDB code: 6OUN) (Bertoletti et al., 2019). For the complex with dCTP, we examined root mean square fluctuations (RMSFs) for all residues in RT during the 24 ns simulation. The RMSF values show minimal fluctuations in the catalytic triad (Asp 110, 185, 186), primer grip region (residues 227–235), and thumb subdomain (residues 237–318). The RMSF analysis suggests that regions of RT important for catalysis and DNA binding are stabilized by the primer-template and dCTP in the active site.

**FIGURE 8:**
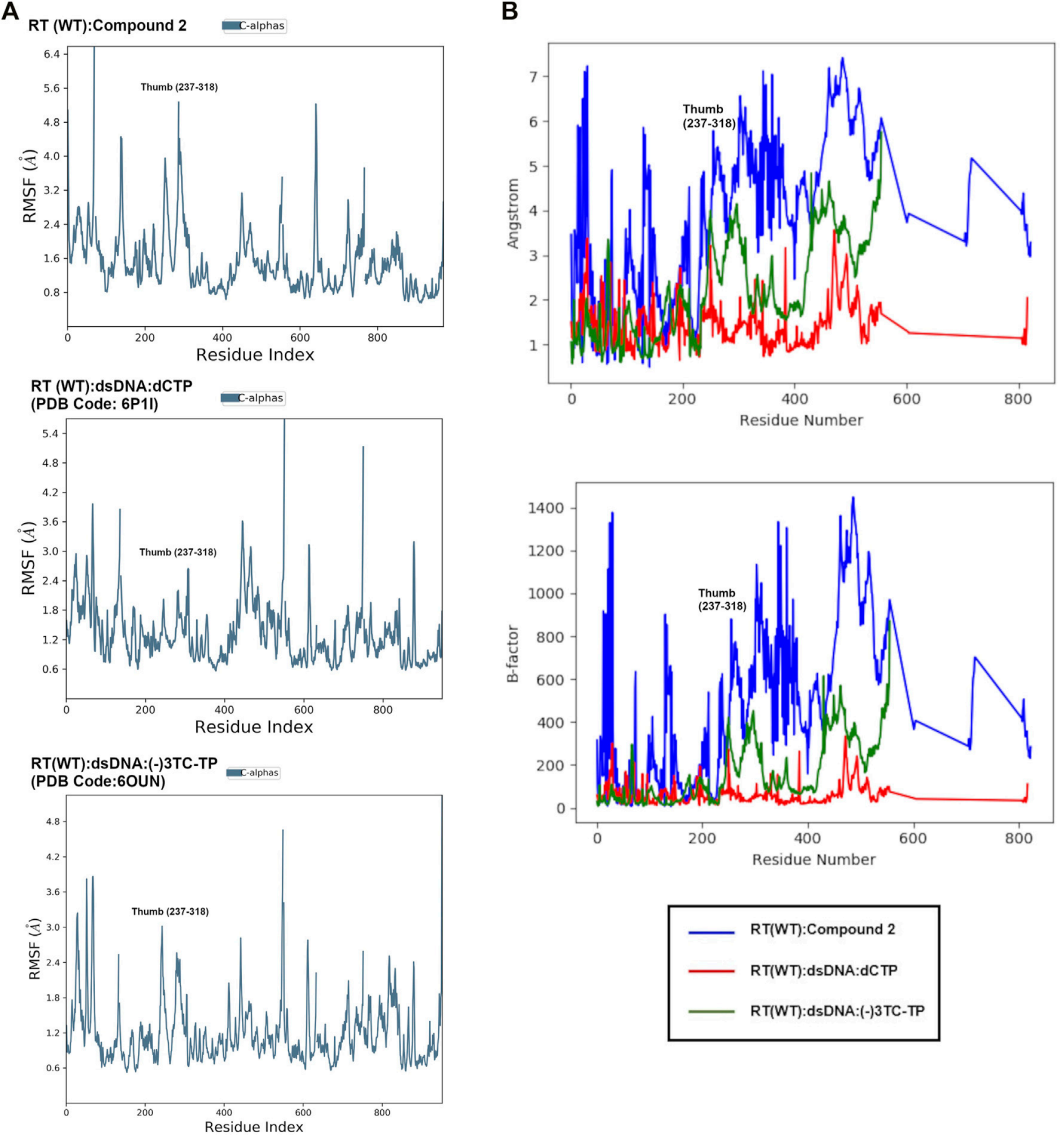
**(A)**: Comparison of root mean square fluctuation (RMSF) values from MD in Å per residue in RT (WT):**2**, RT (WT):dSDNA:dCTP (PDB: 6P1I), and RT (WT):dsDNA:(-)3TC (PDB code: 6OUN). RMSF values in Å were calculated using the RMSF equation in Supplementary Figure S1 using the initial structure (snapshot or frame 0) as reference. The RMSF values represent the average RMSF for each residue during the 24 ns simulation. **(B)**: Overlay of RMSF values for all 3 complexes in Å and scaled to structure B-factors. A noticeable difference in the thumb region of RT is observed when bound to NNRTI compound 2.

In comparison with NNRTIs, we examined the RMSF values for RT (WT) in complex with NNRTI compound 2. RMSF values increase by ∼1–2 Å in the thumb region suggesting an increase in residue flexibility (Figure 8). Most significantly, RMSF values for the thumb subdomain increases by ∼2–5 Å when the NNRTI is bound to RT (WT). Residues in the thumb subdomain appear to have greater fluctuation when the NNRTI is bound compared to the complex with dsDNA primer-template and dCTP (Figure 8).

Interestingly, the thumb subdomain for the (-)-3TC complex are slightly more flexible in the MD simulation, with RMSF values increasing to ∼ 1–2 Å in this region when the NRTI is bound Figure 8). Some residues that show fluctuation are within the subdomain such as L234, H235, P236, and Y318. Both NRTIs and NNRTIs may increase flexibility in the thumb subdomain and destabilize residues that bind to the primer-template.

## Discussion and Conclusion

NNRTIs and NRTIs are commonly used for prevention and treatment of HIV. Structure optimization of NNRTIs is still important for designing new compounds that can overcome physiochemical property and resistance limitations. In our current work, we describe iterations of NNRTI compound optimization guided by crystal structures. We also employed MD simulations to understand the effects of such NNRTIs and NRTIs on RT binding affinity and inhibition of polymerase activity. For all NNRTI complexes examined, the primer grip region is displaced suggesting conformational changes that affect RT polymerase activity. These findings are in accord with our previous kinetic studies to understand how NNRTI binding influences the kinetic reaction pathway (Spence et al., 1995b).

Compound **2** retains affinity for RT (WT), RT (Y181C), and RT (K103N/Y181C) variants. Crystal structures reveal that **2** can maintain most interactions observed in the RT (WT) structure and new interactions with Cys181 of the RT (Y181C) variant are present. The addition of the propoxy linked morpholine also provides potential new interactions while improving solubility. Compounds such as compound **3** and **4** that replace the common cyanovinyl moiety in NNRTIs with CN-naphthyl or CN-indolizine groups also gain additional *π- π* interactions.

The MD simulations identified structural areas that may be flexible in RT complexes with NNRTIs or DNA/NRTIs. The MD simulations for compound **1** helped identify key reasons why the compound was not effective for RT (WT) compared to compound **2**. The MD simulations also revealed that flexibility of the thumb subdomain is influenced by whether a nucleotide, NNRTI, or NRTI is bound. NNRTIs such as compound **2** appear to increase flexibility of this region in addition to distorting the primer grip and YMDD motif. NRTI (-)-3TC also causes an increase in flexibility within the thumb subdomain. Future work using hydrogen deuterium exchange (HDX) experiments with catalytic complexes can help guide our understanding of how NRTIs and NNRTIs affect polymerase activity alone or synergistically.

## Data Availability

The datasets presented in this study can be found in online repositories. The names of the repository/repositories and accession number(s) can be found below: https://www.rcsb.org/ 7SO1 7SNZ 7SNP 7SO3 7SO6 7SO4 7SO2.
