# Supplementary material for: Structural Studies and Structure Activity Relationships for Novel Computationally Designed Non-nucleoside Inhibitors and Their Interactions With HIV-1 Reverse Transcriptase
Source: Front Mol Biosci. 2022 Feb 14;9:805187. doi: 10.3389/fmolb.2022.805187 (PMC8882919; doi:10.3389/fmolb.2022.805187)
Supplement: Supplementary file 1 [file DataSheet1.PDF]

Supplementary Material

1 Synthesis and Characterization of Compound 1

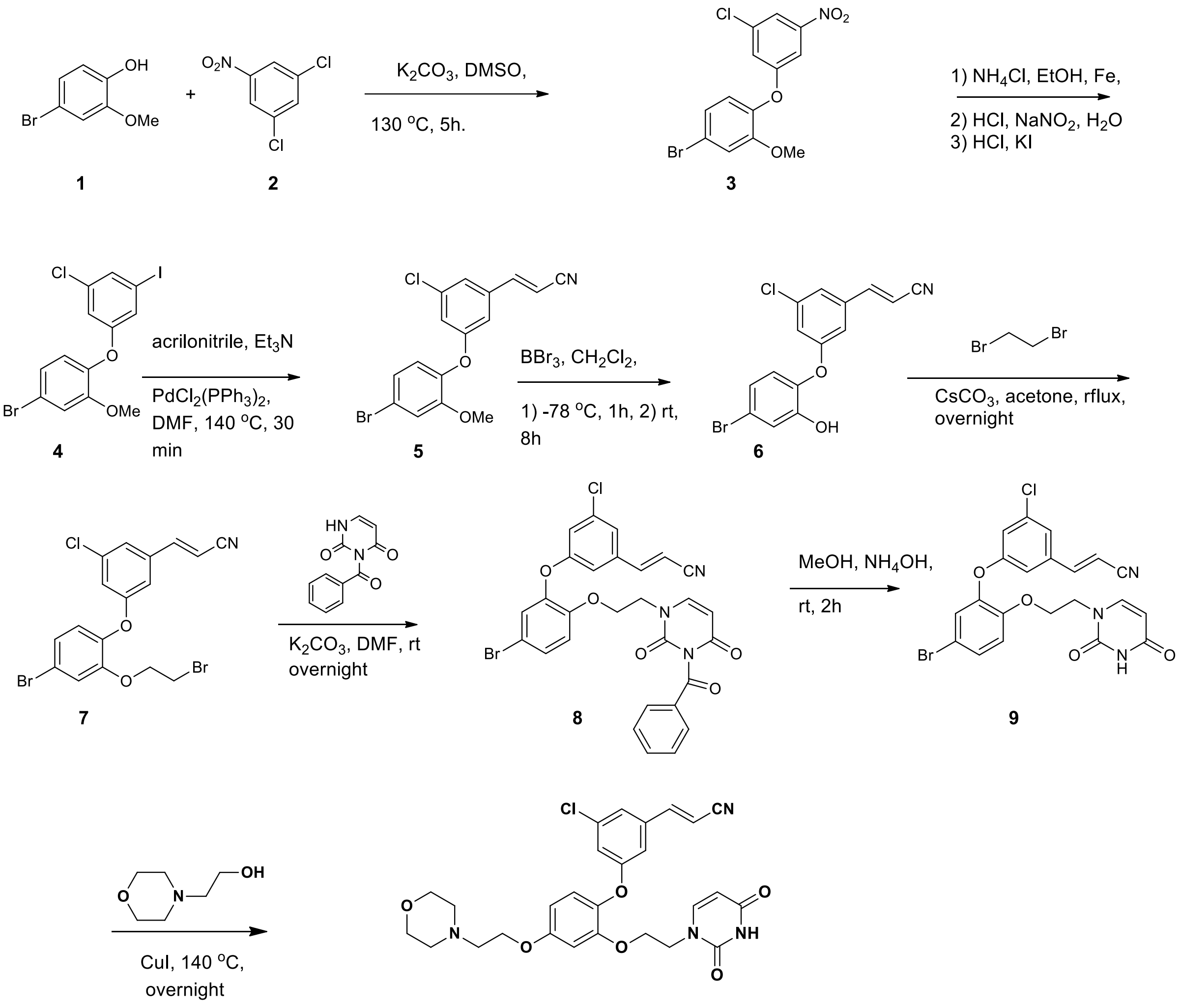

JLJ530

A mixture of 4-bromo-2-methoxyphenol (**1**) (3 g, 14.7 mmol), 1,3-dichloro-5-nitrobenzene (**2**) (2.82 g, 14.7 mmol) in DMSO (10 mL) and anhydrous K_2CO_3 (2.89 g, 21 mmol) was heated at 130 °C for 5 h. The reaction mixture was poured into ice water and extracted with EtOAc (3 x 50 mL). The organic layer was sequentially washed with brine (2 x 75 mL), dried over anhydrous Na_2SO_4, and concentrated in vacuum. The residue was purified by column chromatography to give compound **3**, 4-bromo-1-(3-chloro-5-nitrophenoxy)-2-methoxybenzene (2.3 g, 43%) HR-MS (ES) calcd for $C_{13}H_9BrClNO_2$ [M+1]+ 357.9403, found 357.9392.

Compound **3** (2.26 g, 4.4 mmol), Fe (2.7 g, 35 mmol) and a solution of NH_4Cl (1.10 g in 7.8 mL H2O, 20 mmol) were suspended in 50 mL of EtOH and heated at 75 °C for 1 h. The mixture was allowed to

cool to room temperature; the suspended solid was filtered over celite and the filtrate was concentrated in vacuum. The residue was partitioned between EtOAc and water; combined organic extracts were dried over anhydrous Na_2SO_4 and concentrated in vacuum, to give 3-(4-bromo-2-methoxyphenoxy)-5-chloroaniline (0.9 g, 71 %) The crude of the amino compound (0.9 g, 2.9 mmol) was suspended in concentrated HCl (2.5 mL) at 0 °C and stirred for 30 min. After this period, a solution of $NaNO_2$ in H_2O (0.6 g in 4 mL, 8.8 mmol) was added dropwise. Then the resulting mixture was stirred for 15 min at room temperature. This solution was added over 30 min to a solution of KI (0.95 g, 17.2 mmol) in concentrated HCl (7.0 mL) at 60 °C. After addition, the mixture was heated at 80 °C for 30 minutes. The mixture was allowed to cool to room temperature before addition of brine. The solution was extracted with ethyl acetate, dried over anhydrous $MgSO_4$ and evaporated in vacuo. The crude product was purified by column chromatography (SiO_2, hexane/EtOAc 100: 0 to 80:20) to give 4-bromo-1-(3-chloro-5-iodophenoxy)-2-methoxybenzene (**4**) (1.1 g, 76 %). ^{1}H NMR (500 MHz, $CDCl_3$) 7.21 (d, J = 6.01 Hz, 1H), 7.12 (s, 1H), 7.09 (m, 1H), 6.98 (d, J = 1.78 Hz, 1H), 6.90-6.87 (m, 2H), 3.73 (d, J = 4.47 Hz, 3H).

Acrylonitrile (0.8 mL, 13.5 mmol) was added to a mixture of **4** (1 g, 2.2 mmol), Et_3N (1.1 mL, 8.4 mmol), $PdCl_2(PPh_3)_2$ (0.1g, 0.12 mmol), and DMF (10.0 mL) under a nitrogen atmosphere at room temperature. The mixture was heated at 140 °C for 30 min. The reaction mixture was quenched with water and the mixture was extracted with EtOAc (3 x 50 mL). The organic layer was sequentially washed with brine (2 x 75 mL), dried over anhydrous Na_2SO_4, and concentrated in vacuo. The residue was purified by column chromatography (SiO_2, hexane/EtOAc 80:20) to give (*E*)-3-(3-(4-bromo-2-methoxyphenoxy)-5-chlorophenyl)acrylonitrile (**5**) (0.42 g, 53 %) ^{1}H NMR (400 MHz, $CDCl_3$) δ 7.30 (dd, J = 4.47, 7.59 Hz, 2H), 7.11 – 7.09 (m, 2H), 6.99 – 6.94 (m, 3H), 5.82 (d, J = 16.64 Hz, 1H), 3.82 (s, 3H). HR-MS (ES) calcd for $C_{16}H_{11}BrClNO_2$ $[M+1]^+$ 363.9662 found 363.9672

A solution of BBr_3 (5.0 mmol, 1M in CH_2Cl_2) was added dropwise to a solution of **5** (1.8 mmol) in dry CH_2Cl_2 (4.0 mL) under N_2 at -78 °C. The reaction mixture was stirred at this temperature for 1 h. After this period, the reaction was allowed to warm to room temperature and stirred for 8 h. After completion, the solution was quenched with ice-water, the solvent was removed in vacuo and the residue was extracted with CH_2Cl_2 and washed with a solution of $NaHCO_3$. The combined organic layer was washed with brine, dried over anhydrous Na_2SO4 and concentrated in vacuo. The residue was purified by column chromatography (SiO_2, Hexane/EtOAc 80:20) to give (*E*)-3-(3-(4-bromo-2-hydroxyphenoxy)-5-chlorophenyl)acrylonitrile (**6**) (0.5 g, 75%) HR-MS (ES) calcd for $C_{15}H_9BrClNO_2$ $[M+1]^+$ 349,9505 found 349,9499

Compound **6** (0.47g, 1.3 mmol, Cs_2CO_3 (0.54 g, 1.3 mmol), 1,2 dibromoethane (0.9 g, 5.3 mmol) in acetone was stirred at reflux overnight. The reaction mixture was filtered, and the filtrate was concentrated under vacuum. The crude product was purified by column chromatography (SiO_2, hexane/EtOAc 70:30) to give (*E*)-3-(3-(4-bromo-2-(2-bromoethoxy)phenoxy)-5-chlorophenyl)acrylonitrile (**7**) (0.50 g, 81 %) ^{1}H NMR (500 MHz, CDCl3) δ 7.40 – 7.37 (m, 1H), 7.28 (t, J = 1.44 Hz, 1H), 7.07 (dd, J = 5.28, 8.26 Hz, 2H), 6.99 (d, 8.26 Hz, 1H) 6.18 (dt, J = 2.22, 4.14 Hz, 2H), 5.75 (d, J = 16.61 Hz, 1H), 4.16 (t, J = 6.33 Hz, 2H), 3.38 (t, J = 6.32 Hz, 2H).

Compound **7** (0.32 g, 0.7 mmol) was treated with 3-benzoylpyrimidine-2,4(1H,3H)-dione (0.18 g, 0.12 mmol) and K_2CO_3 (0.18g, 0.84 mmol) in DMF (2 mL). The reaction mixture was stirred at room temperature for 24 h. Then, was poured into a solution of NH_4Cl and extracted with EtOAc (3 x 20 mL). The organic layer was sequentially washed with brine (2 x 15 mL), dried over anhydrous Na_2SO_4, and concentrated in vacuo. The crude product (**8**) was dissolved in MeOH (5 mL) and NH_4OH (4.0 mL) was added. The reaction mixture was stirred at room temperature for 2 h. Then, the solvent was

removed in vacuum and purified by column chromatography (SiO_2, hexane/EtOAc 20:80) to give (*E*)-3-(3-(5-bromo-2-(2-(2,4-dioxo-3,4-dihydropyrimidin-1(2H)-yl)ethoxy)phenoxy)-5-chlorophenyl)acrylonitrile (**9**) (0.24 g, 58%) ^{1}H NMR (500 MHz, $CDCl_3$) δ 8.30 (s, 1H), 7.58 (s, 1H), 7.15 (d, J= 16.10 Hz, 1H),7.01-6.95 (m, 3H), 6.77 (dd, J = 2.64, 8.11 Hz, 2H), 6.64 (s, 1H), 5.81 (d, J = 16.10 Hz, 1H), 5.67 (d, J = 7.90 Hz, 1H), 4.14 (d, J = 4.08 Hz, 2H), 3.94 (s, 2H).^{13}C NMR (126 MHz, CDCl3) δ 160.12, 158.01, 149.61, 147.18, 144.19, 134.96, 122.67, 122.0, 121.02, 117.01, 114.05, 111.46, 100.45, 98.25, 76.25, 76.00, 75.74, 65.60, 47.28, 28.68. HR-MS (ES) calcd for $C_{21}H_{15}BrClN_3O_4$ $[M+1]^+$ 487,9934, found 487,9890.

Compound **9** (0.1 g, 0.2 mmol) and 2-morpholinoethan-1-ol (1 g, 6 mmol) were placed into a round bottom flask containing CuI (0.07 g, 0.038 mmol). The obtained mixture was heated at 140 °C for 4 days. The reaction mixture was cooled after completion of the reaction and the catalyst was separated by filtration. The filtrate was vaporized on a rotary evaporator and the crude product was purified by column chromatography (SiO_2, hexane/EtOAc 10:90) to give (*E*)-3-(3-chloro-5-(2-(2-(2,4-dioxo-3,4-dihydropyrimidin-1(2H)-yl)ethoxy)-4-(2-morpholinoethoxy)phenoxy)phenyl)acrylonitrile (**JLJ530**) ^{1}H NMR (500 MHz, Acetone-d_6) δ δ 9.93 (s, 1H), 7.39 (d, *J* = 16.9 Hz, 1H), 7.23 (s, 1H), 7.11 (d, *J* = 7.8 Hz, 2H), 6.84-6.72 (m, 3H), 6.51 (d, *J* = 2.4 Hz, 1H), 6.19 (d, *J* = 16.8 Hz, 1H), 5.04 (d, *J* = 8.0 Hz, 1H), 4.36 (d, J = 4.1 Hz, 2H), 4.22 – 4.07 (m, 2H), 3.97 – 3.70 (m, 2H), 3.17 (t, *J* = 6.4 Hz, 2H), 3.08 (t, *J* = 7.3 Hz, 4H), 2.56 – 2.47 (m, 4H). ^{13}C NMR (125 MHz, Acetone-d_6) 163.55, 159.84, 151.16, 149.00, 145.69, 136.39, 133.68, 132.47, 129.06, 127.76, 124.11, 120.80, 118.09, 117.83, 112.47, 101.70, 98.94, 76.83, 66.61, 65.7, 55.16, 53.62, 38.84, 31.31, 30.11. HR-MS (ES) calcd for $C_{27}H_{27}ClN_4O_6$ $[M+1]^+$ 539,1619 found 539,1610

2 Validation Reports for Molecular Dynamics (MD) Simulations for Compounds 1

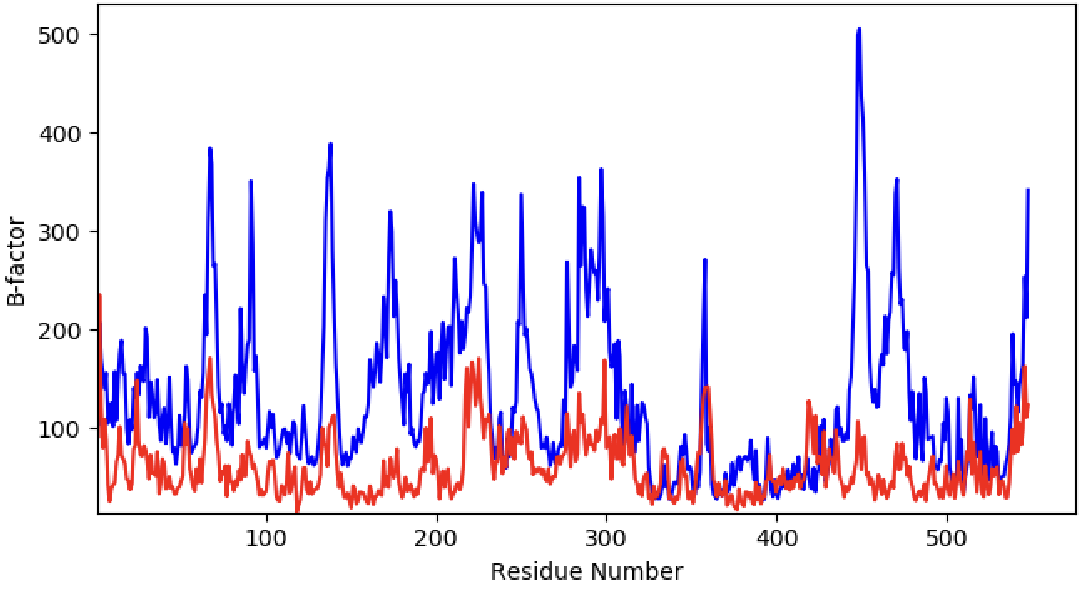

Supplementary Figure 1. Root mean square fluctuations (RMSF) values from MD simulation for **Compound 1**. Residue index or position number in RT is plotted against RMSF scaled to B-factors.

Key: RMSF scaled to B-factors (blue); Structure B-factors (red). RMSF values were calculated using the following equation:

$$RMSF_i = \sqrt{\frac{1}{T}\sum_{t=1}^{T} < (r_i'(t)) - r_i(t_{ref}))^2 >}$$

where T is the trajectory time over which the RMSF is calculated, t_{ref} is the reference time, r_i is the position of residue i; r' is the position of atoms in residue i after superposition on the reference, and the angle brackets indicate that the average of the square distance is taken over the selection of atoms in the residue.

As specified by Schrodinger, N and C-termini may fluctuate more than other regions of the protein.

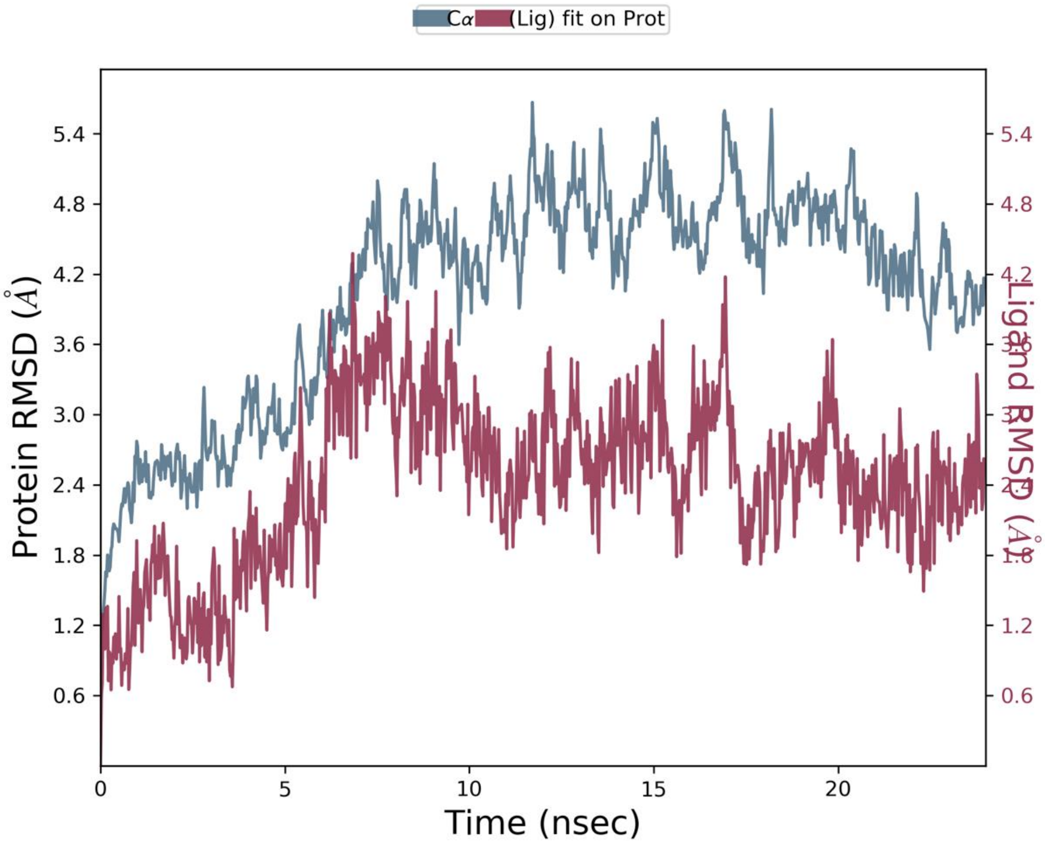

Supplementary Figure 2. Root mean square deviation (RMSD) values versus time from MD simulation for **Compound 1 (red)** and the C**α backbone of RT (WT) (blue)**. RMSD values were calculated using the following equation:

$$RMSD_x = \sqrt{\frac{1}{N}\sum_{i=1}^{N}(r_i'(t_x)) - r_i(t_{ref}))^2}$$

where N is the number of atoms in the atom selection; t_{ref} is the reference time, (typically the first frame is used as the reference and it is regarded as time t=0); and r' is the position of the selected atoms in frame x after superimposing on the reference frame, where frame x is recorded at time t_x. The procedure is repeated for every frame in the simulation trajectory.

As specified by Schrodinger, the RMSD analysis in the Desmond software is used to show that the protein has equilibrated in the simulation. Changes of 1-3 Å are acceptable for globular proteins; RMSD values should stabilize around a fixed value to indicate convergence.

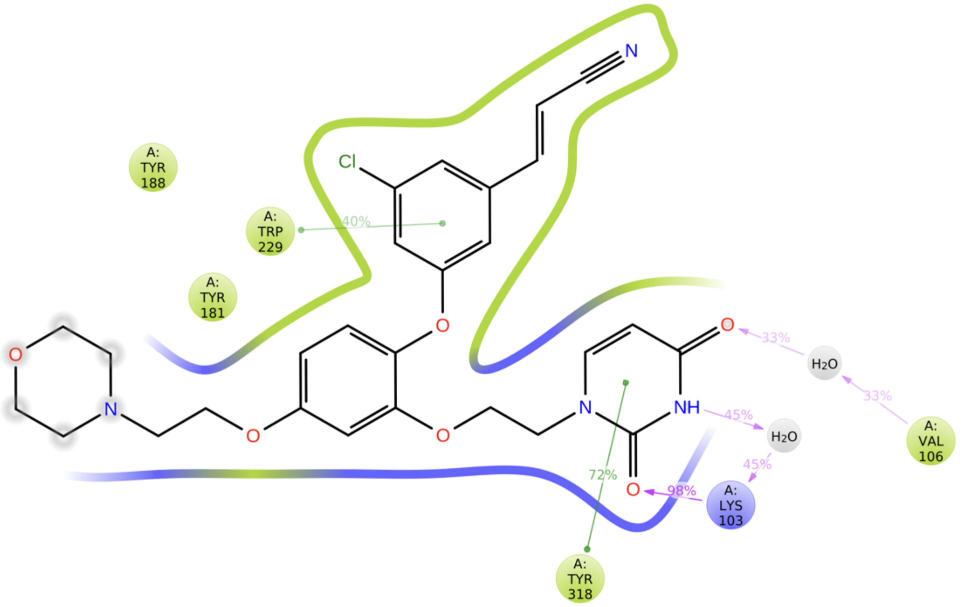

Supplementary Figure 3. 2D Schematic of protein-ligand contacts made between RT (WT) and compound 1 in MD simulation. Pink arrows represent hydrogen bond interactions and green lines represent aromatic stacking interactions. Percentages shown for each interaction represent the percent the interaction is observed in the MD snapshots. Purple arrows represent hydrogen bonds; green lines represent π-π interactions.

3 Validation Reports for Molecular Dynamics (MD) Simulations for Compounds 2

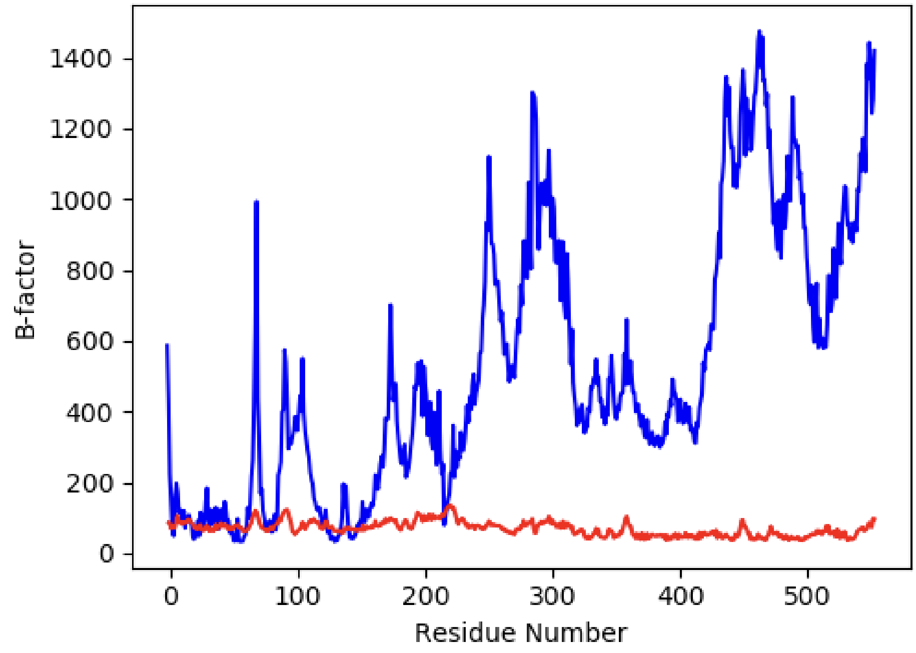

Supplementary Figure 4. Root mean square fluctuations (RMSF) values from MD simulation for **Compound 2**. Residue index or position number in RT is plotted against RMSF scaled to B-factors. Key: RMSF scaled to B-factors (blue); Structure B-factors (red). RMSF values were calculated using the same equation specified in supplementary figure 1 caption.

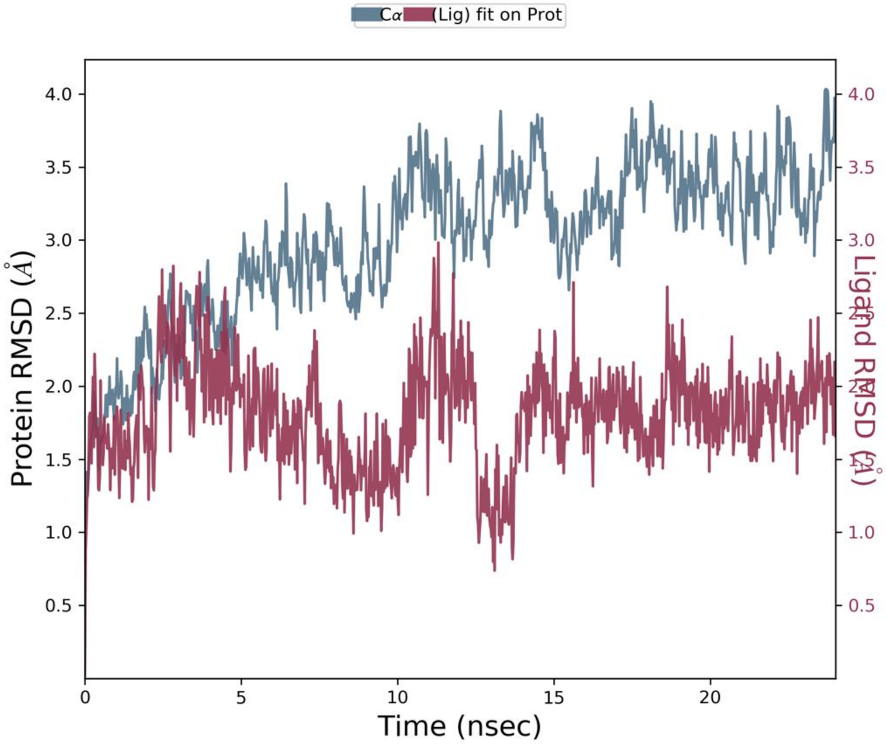

Supplementary Figure 5. Root mean square deviation (RMSD) values versus time from MD simulation for **Compound 2 (red)** and the Cα **backbone of RT (WT) (blue)**. RMSD values were calculated using the same equation specified in supplementary figure 2 caption.

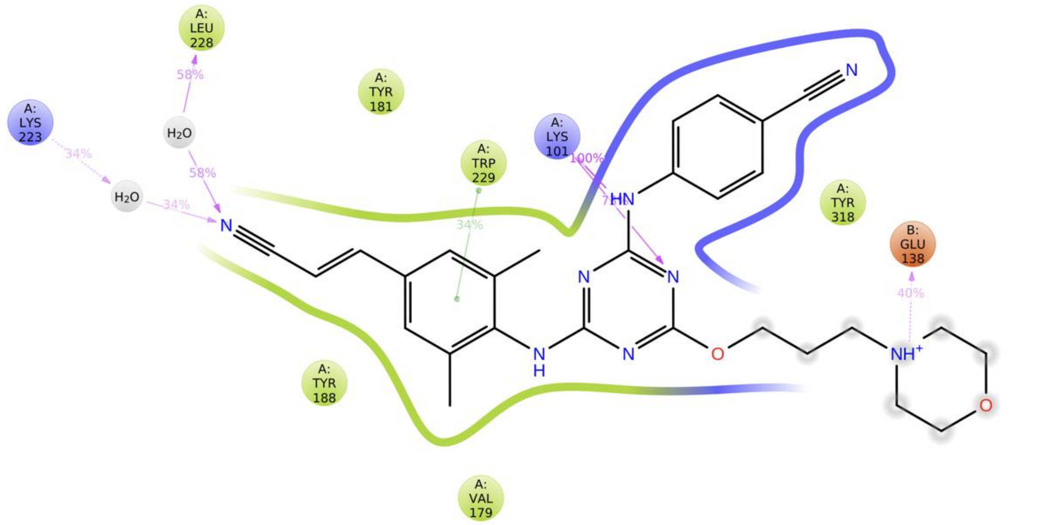

Supplementary Figure 6. 2D Schematic of protein-ligand contacts made between RT (WT) and compound **2** in MD simulation. Pink arrows represent hydrogen bond interactions and green lines represent aromatic stacking interactions. Percentages shown for each interaction represent the percent the interaction is observed in the MD snapshots. Purple arrows represent hydrogen bonds; green lines represent π-π interactions.

4 Validation Reports for Molecular Dynamics (MD) Simulations for RT(WT):dsDNA:dCTP (PDB code: 6P1I)

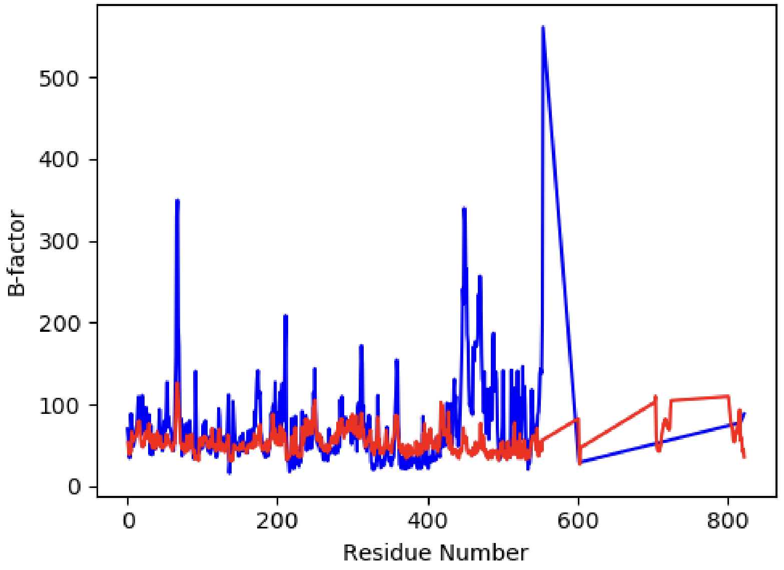

Supplementary Figure 7. Root mean square fluctuations (RMSF) values from MD simulation for RT(WT):dsDNA:dCTP. Residue index or position number in RT is plotted against RMSF scaled to B-factors. Key: RMSF scaled to B-factors (blue); Structure B-factors (red). RMSF values were calculated using the same equation specified in supplementary figure 1 caption.

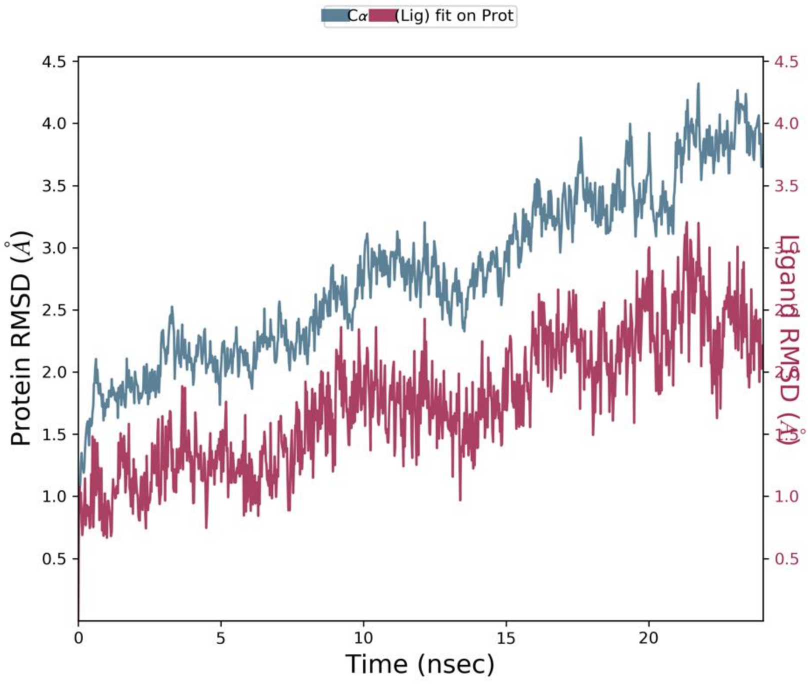

Supplementary Figure 8. Root mean square deviation (RMSD) values versus time from MD simulation for dCTP **(red)** and the Cα **backbone of RT (WT) (blue)**. RMSD values were calculated using the same equation specified in supplementary figure 2 caption.

6 Validation Reports for Molecular Dynamics (MD) Simulations for RT(WT):dsDNA:(-)3TC-TP (PDB code: 6OUN)

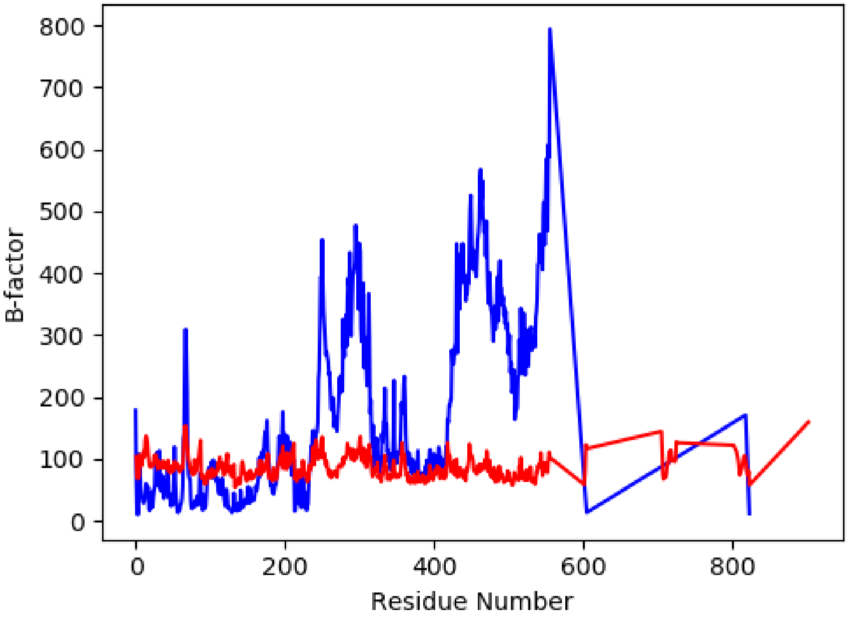

7 **Supplementary Figure 9.** Root mean square fluctuations (RMSF) values from MD simulation for RT(WT):dsDNA(-)-3TC-TP. Residue index or position number in RT is plotted against RMSF scaled to B-factors. Key: RMSF scaled to B-factors (blue); Structure B-factors (red). RMSF values were calculated using the same equation specified in supplementary figure 1 caption.

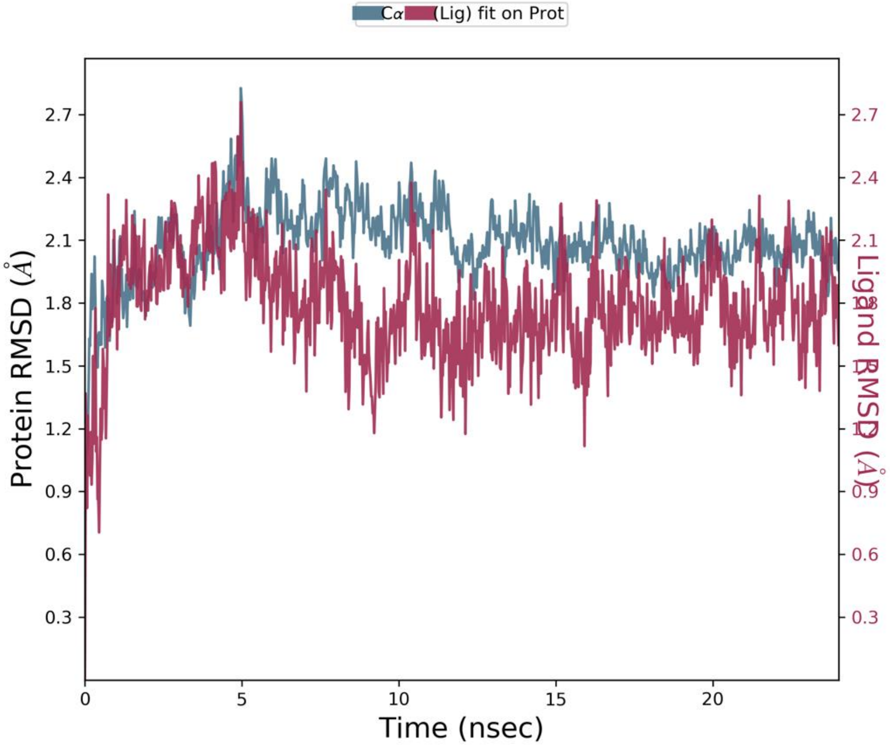

Supplementary Figure 10. Root mean square deviation (RMSD) values versus time from MD simulation for (-)-3TC-TP **(red)** and the Cα **backbone of RT (WT) (blue)**. RMSD values were calculated using the same equation specified in supplementary figure 2 caption.

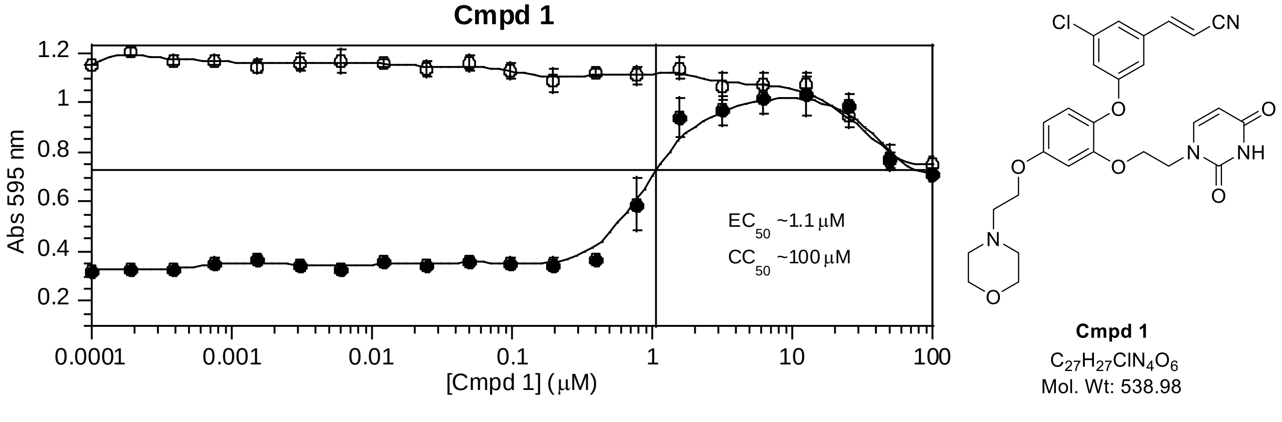

Supplementary Figure 11. Representative data for assessing antiviral activity of Compound **1** using the MTT assay. Open circles examine the cellular cytotoxicity (CC_{50}) in MTT cells in the absence of virus. Filled circles examine the antiviral efficacy (EC_{50}) of Compound **1**. Triplicate samples are analyzed at each concentration of compound.
